# Supplementary material for: Are carbon emissions trading and green financial instruments synergistic? -Comprehensive quantitative research based on content analysis
Source: PLoS One. 2024 Mar 7;19(3):e0298601. doi: 10.1371/journal.pone.0298601 (PMC10919601; doi:10.1371/journal.pone.0298601)
Supplement: S1 Data — (ZIP) [file pone.0298601.s001.zip › Data/地级市Z_F工作报告.zip!/╣≤╓▌/┴∙┼╠╦«╩╨/└┤╫╘╣1⁄2╓┌║┼í╛┬φ┐╦╩2╛▌═°í┐_2015.docx]

各位代表：

　　现在，我代表市人民政府向大会作工作报告，请予审议，并请各位政协委员和列席的同志提出意见。

　　一、2014年工作回顾

　　过去一年，在市委的坚强领导下，在市人大、市政协的监督和支持下，我们认真贯彻落实习近平总书记系列重要讲话精神和中央、省委省政府各项决策部署，高举“发展、为民”的旗帜，坚持主基调主战略，牢牢守住发展、生态、安全三条底线，积极主动适应新常态，统筹做好稳增长、调结构、促改革、惠民生、防风险各项工作，较好地完成了全年各项目标任务，保持了经济社会持续健康发展。

　　一是经济运行平稳。全市生产总值突破千亿元大关，人均生产总值实现5876美元，高于全省平均数。各项经济社会发展指标稳步增长，经济社会风险可控，经济运行处于合理区间。二是转型步伐加快。三次产业结构从6.6：57.1：36.3调整到7.2：54.4：38.4，产业投资比重达到45%。山地特色现代农业发展势头强劲。煤炭资源就地转化率提高15个百分点。非税收入占比下降13.85个百分点，财税结构逐步优化。三是发展活力增强。市场主体、注册资本金分别增长12%、37.7%。民营经济占比达到52%。县域经济占比达到68.1%。招商引资实际到位资金713亿元，增长43.6%。四是发展环境优化。综合交通建设取得历史性突破，月照机场建成通航；六六高速建成通车，我市成为全省第二个县县通高速的市州。水利工程、生态建设全面推进，发展基础不断夯实。五是民生持续改善。减少贫困人口14.55万人。新增就业10.02万人。农村和城镇居民人均可支配收入分别达到6791元、21168元，增长12.9%、11.1%。全面小康实现程度达到77.4%。

　　一年来，我们重点抓了以下九个方面工作：

　　（一）坚持以加快发展为要务，促进经济平稳增长。狠抓目标管理，细化分解目标任务，强化经济运行调度，推动经济工作落实。大力实施工业“百千万”工程，深入开展“双服务”大行动，稳定经济运行。全市生产总值完成1042.73亿元，增长14.1%；50万元以上固定资产投资完成1336.27亿元，增长26.7%；规模以上工业增加值完成456.36亿元，增长11.5%；公共财政预算收入完成128.74亿元，增长4.2%；社会消费品零售总额完成236.9亿元，增长13.2%；金融机构存贷款余额分别完成817.1亿元、701.2亿元，增长8.3%、16.9%。

　　（二）坚持以转型升级为导向，着力优化产业结构。加快产业结构调整步伐，推动三次产业转型升级、融合发展。一产转型迈出新步伐。全面实施农业特色产业“3155工程”，完成特色产业种植88.98万亩；调减粮食作物种植面积30万亩；粮经比从51：49调整到39：61。现代高效农业示范园区建设加快推进，新增11个市级园区为省级园区，全市省级园区达26个；水城米萝猕猴桃产业园区、六枝郎岱农业园区在全省绩效考评中分别排第一位和第五位。盘县刺梨果脯等6个特色农产品获国家地理标志产品保护。我市荣获“中国野生猕猴桃之乡”、“中国野生刺梨之乡”、“中国红豆杉之乡”称号。省级、市级龙头企业分别达33家、126家，新增农民专业合作社266家。二产升级取得新成效。产业园区产值突破千亿元，完成1028.8亿元，增长17.6%。黔桂天能煤电化循环经济园区建成，开发苯加氢、LNG等19个煤焦化下游产品。煤层气开发利用率达到33.3%。红豆集团生物制药、制衣项目和红豆杉基地开工建设，富士康精密电子制造等一批战略性新兴产业项目建成投产。品三商砼公司、安凯达公司等新型建筑企业在“新三板”挂牌上市，实现我市民营企业上市零的突破。三产优化实现新突破。召开了全市第一届旅游文化产业发展大会，牂牁江、妥乐古银杏、野玉海等一批特色旅游景区实现升级提档，玉舍滑雪场填补了我省冬季旅游项目空白，旅游收入增速位列全省第一，“贵州屋脊·中国凉都”知名度和美誉度不断提升。加大“引金入市”力度，引进了招商银行、贵阳银行等金融机构。商贸物流、文化体育、康体养生等现代服务业加快发展。

　　（三）坚持以项目建设为抓手，持续扩大投资规模。紧紧围绕“5个100工程”、交通水利基础设施、社会事业等重点领域，全力推进重大工程、重大项目投资建设。全年集中新开工项目1296个，总投资2086.3亿元，完成投资747.5亿元。加强项目策划包装，全市项目库总投资达1.83万亿元；纳入预安排国家重大工程项目313个，总投资953亿元。市属投融资公司融资到位资金105.21亿元，建立政府性债务风险化解机制；完善土地管理长效机制，加强土地收储和供给。推进项目审批、投资审计规范化、制度化建设，出台政府投资建设项目管理、前置审计等制度；审计政府性投资项目110个，审减金额8.97亿元。

　　（四）坚持以三地同城为引擎，推动城乡统筹发展。全面推进山地特色新型城镇化，城镇化率达到44.5%。第四轮城市总体规划修编获省城规委审查通过。启动市中心城区控规全覆盖编制工作，完成棚户区改造等33个专项规划、56个乡镇总体规划和319个村庄整治规划。加快推进钟山、水城、红桥同城发展，完成内环快线主体工程，水西南路建成通车，机场高速加快建设，棚户区改造、钟山大道和人民广场地下人防工程、水城河综合治理工程等项目全面启动实施。完成市中心城区9个农贸市场升级改造。盘县、六枝城市路网建设加快，城市功能日趋完善，城市拉力持续增强。大力推进示范小城镇建设，建成“8+X”项目32个，新增省级示范小城镇1个。“四在农家·美丽乡村”基础设施建设六项行动计划完成投资39亿元。深入推进“四创”活动，城市精细化管理水平进一步提升。盘县通过“国家卫生县城”评估；我市获“全省文明城市”称号，水城县、钟山区、盘县获“全省文明县城”称号。

　　（五）坚持以交通水利为重点，大力破解发展瓶颈。实施交通建设“三年会战”，安六城际铁路开工建设，沪昆客运专线境内段建设加快，六盘水火车站新站房投入使用。杭瑞高速毕节至都格段、盘兴高速建设加快，都香高速六盘水至威宁段、纳晴高速境内段前期工作有序推进；开工建设一批景区景点旅游公路和重要节点公路项目，提前启动实施“十三五”规划通村油（水泥）路1734公里。推进水利建设“三大会战”，纳入“三位一体”规划的骨干水源工程开工14个；黔中水利枢纽一期主体工程和双桥水库基本建成，大中型水库、水电站下闸蓄水6个，排全省第一；花岩洞应急取水工程并网供水。开发利用地下水机井232口，解决26.83万农村人口饮水安全问题。加强电力通信设施建设，建成10千伏以上项目313个、变电站16座，新增线路1994公里，实现市中心城区220千伏电网全覆盖；市县乡中心区域实现4G网络全覆盖。

　　（六）坚持以改革开放为动力，不断激发发展活力。全面推进2批104个重点改革专题。资源市场化体制改革取得重要突破。公共资源交易项目实现“应进必进”。矿业权招拍挂出让制度全面推行。统筹城乡发展体制改革步伐加快。开展土地承包经营权确权登记颁证，有序推进水城县农村产权确权整县试点。盘县被列为全省城乡总体规划“多规融合”试点县。生态文明建设体制改革不断深入。组建市生态旅游委，将生态环保纳入各县区工作目标管理控制性指标考核。积极推动国家生态功能区补偿改革试点前期工作。国有企业改革改制不断推进。基本完成128家市属国有企业清产核资工作。组建8家市属投融资公司。采取PPP模式完成市燃气总公司改革改制。出台国有企业用人、资产收益管理等制度。水城矿业集团产值突破百亿元，全市实现百亿元产值的企业达3家。扶贫开发机制改革有效推进。认真落实精准扶贫和“六个到村到户”，完成52个贫困村“整村推进”和15个贫困乡镇“减贫摘帽”。出台财政扶贫资金有偿使用滚动发展实施办法、财政专项扶贫资金管理办法、金融扶贫实施方案。财税金融改革稳步实施。加强政府性债务分类管理，建立政府性债务风险化解准备金。强化市级财政支出绩效评价，规范党政机关差旅费、会议费及“三公”经费等支出。出台完善金融市场体系建设实施意见。教育领域综合改革扎实开展。出台加快教育改革发展的实施意见。启动基本普及15年教育计划。全面深化职校进园区办学模式。行政管理体制改革不断深化。完成市、县两级政府机构改革。加快推进乡镇行政区划调整。积极推进政府向社会力量购买服务工作。继续压缩行政经费5%共4470万元用于教育“9+3”计划。开放型经济体制改革全面推进。完善招商引资项目考核办法，推行招商引资项目全程代办和并联审批制度；全方位开展招商引资，引进华润、富士康、红豆集团等500强企业进驻六盘水。加强交流合作，与瑞士洛桑酒店管理学院、贵阳市、黔西南州、贵阳海关签订战略合作框架协议。社会领域改革深入推进。引进汉方集团参与凉都人民医院建设和办医；启动县级公立医院改革试点。全面实施“单独二孩”政策。制定科技计划项目绩效评估管理规定。出台加强城市基层管理推进社区建设的实施意见。

　　（七）坚持以生态文明为引领，牢牢守住生态底线。大力发展循环经济，编制《六盘水市建设国家循环经济示范城市发展规划》并上报国家发展改革委。加强资源循环利用，工业固体废弃物综合利用率达到68%。组织开展森林保护“六个严禁”执法行动，实施新一轮退耕还林工程20万亩，完成营造林45.37万亩，治理石漠化53.5平方公里、水土流失55.58平方公里，森林覆盖率达到45.4%。明湖国家湿地公园荣获“最美生态湿地公园”称号；牂牁江国家湿地公园、月亮河森林公园获国家林业局批准。成功举办2014年生态文明贵阳国际论坛六盘水水生态文明与水生态基础设施分论坛。深入实施“十二件环保实事”，严格执行环境监管“六个一律”，城市污水、生活垃圾无害化处理率分别达到75.7%和71%。从去年6月1日起定期发布PM2.5等六个参数的空气质量指数，优级天数达62天，良级天数达137天。三岔河河长制考核排名全省第一。扎实推进节能减排，淘汰落后产能89万吨，单位生产总值能耗比上年下降2.49%，主要污染物排放总量控制在省下达指标范围内。

　　（八）坚持以改善民生为目的，统筹发展社会事业。扎实办好“十件民生实事”，完成投资79亿元，占计划的120%。深入实施教育“9+3”计划，完成学前教育工程26个，建设寄宿制学生宿舍15.73万平方米，开工建设高中教育城，加快建设以朵职教城、六盘水师院二期、六盘水职院二期工程。开工建设凉都人民医院，建成12个乡镇卫生院、44个村卫生室；完成全市乡、村两级卫生计生机构整合；扎实推进人口计生“双诚信双承诺”工作和“三按月”绩效管理，完成“三降一升”目标任务。

　　成功举办获得“银牌赛事”的夏季国际马拉松赛等凉都消夏文化节系列活动，成功承办全省第五届残疾人运动会等重大赛事活动；组建贵州磅礴传媒集团。启动国家级创业型城市创建工作；落实“3个15万元”政策，新增微型企业3006户，带动就业1.71万人。引进高层次和急需特殊人才1280人。城乡居民最低生活保障标准分别提高10.25%和10.17%，认真落实临时救助制度。完成4大省属国有企业职工社会保险移交办理工作，全市基本社会保险覆盖率达到90%以上。新农合参合人数226万人，参合率达到99.06%。改造农村危房20700户，建成保障性住房9114套，完成住房公积金支持保障房建设试点城市项目贷款6.4亿元。深入开展安全生产大检查、大整治、大防范，认真制定并落实煤矿安全生产“8个100%”措施，集中开展打非治违“六打六治”专项活动，安全生产事故起数和死亡人数分别下降10.83%和6.12%。“平安凉都”建设深入推进，钟山区获“全国和谐社区建设示范城区”荣誉称号。妇女、儿童、老龄、残疾人等事业积极发展，工会、共青团、民族、宗教、外事、侨务、对台、档案、气象、人防、红十字、地方志等工作取得新成效。

　　（九）坚持以转变职能为核心，切实提高行政效能。深入开展党的群众路线教育实践活动，严格执行中央八项规定和省、市十项规定。出台《市政府议事规则》等制度，进一步规范政府工作。坚持依法行政，科学民主决策，公布市级36个行政机关权力清单。取消和下放行政许可审批事项162项，推行“错时、延时、上门、订单、预约”五项服务和“领导进中心”、“局长进大厅”等活动，行政审批服务事项办理时限压缩率达到65%。自觉接受人大法律监督、政协民主监督，办好人大代表建议和政协委员提案。加强行政监察和审计监督，认真落实党风廉政建设主体责任，勤政廉政建设取得新成效。

　　各位代表！回顾过去一年的工作，我们深刻体会到：必须牢牢把握经济新常态这个发展大势，坚决贯彻落实中央、省委和市委的决策部署，只要中央有要求、省委有决策、市委有部署，市政府就有行动、有落实；必须牢牢把握加快发展这个第一要务，坚持主基调、实施主战略，不动摇、不懈怠，推动经济社会又好又快、更好更快发展；必须牢牢把握维护稳定这个第一责任，加强社会治理、化解矛盾纠纷、抓好安全生产，营造加快发展的良好环境；必须牢牢把握保障改善民生这个根本，坚持把人民群众对美好生活的期盼作为奋斗目标，着力提高群众生活水平；必须牢牢把握生态建设这条底线，坚持生态产业化、产业生态化，实现百姓富与生态美的有机统一；必须牢牢把握转变作风这个关键，大力整治“四风”突出问题，以踏石留印、抓铁有痕的精神推进政府自身改革，不断提高政府治理能力和水平。这些基本经验，我们将在今年的政府工作中继续坚持。

　　各位代表！过去一年的成绩来之不易。这是市委坚强领导的结果，是市人大、市政协监督支持的结果，是全市各族干部群众团结奋斗和各方大力支持的结果。在此，我代表市人民政府，向全市各族人民，向人大代表、政协委员，向各民主党派、工商联、无党派人士和各人民团体，向驻市人民解放军、武警消防官兵、政法公安干警，向所有关心和支持我市改革开放和现代化建设的各界人士，表示衷心的感谢和诚挚的谢意！

　　在充分肯定成绩的同时，我们也清醒地看到存在的困难和问题。一是经济下行压力加大，受要素成本不断上升、工业产品价格持续下跌两头挤压，全市煤、钢、电、铝等传统工业增速放缓，保持经济较快增长的任务艰巨；二是精准扶贫不到位，贫困人口稳定增收保障不强，公共服务和社会保障体系不完善，安全生产形势依然严峻，保障和改善民生的任务艰巨；三是生态环境脆弱，水土流失和石漠化治理难度大，生态工程建设和环境保护任务重，节能减排压力大，守住生态底线的任务艰巨；四是政府性债务压力加大，煤炭、钢铁和房地产等行业存在经济风险和社会风险双重压力，防范化解风险的任务艰巨；五是一些干部本领不强、作风不实、担当不够、执行不力，营造良好发展环境的任务艰巨。对存在的这些问题，我们将高度重视，坚持用发展的办法、改革的举措和扎实的作风认真加以解决。

　　二、2015年工作目标和主要任务

　　2015年是全面深化改革的关键之年，是全面推进依法治国的开局之年，是全面完成“十二五”规划的收官之年。今年政府工作的总体要求是：以邓小平理论、“三个代表”重要思想、科学发展观为指导，深入学习贯彻习近平总书记系列重要讲话精神，全面贯彻中央和省委各项决策部署，紧紧围绕建设国家循环经济示范城市、区域中心城市、国际标准旅游休闲度假城市、大健康旅游目的地城市目标，高举循环经济旗帜，守住发展、生态、安全三条底线，坚持主基调、主战略，主动适应新常态，主动抢抓新机遇，主动实现新作为，以上率下、把握大势、统筹全局、着眼民生、提速转型，推动政治、经济、文化、社会、生态文明开创新局面，为实现同步小康奠定坚实基础。

　　今年经济社会发展的主要预期目标是：地区生产总值增长14%以上；公共财政预算收入增长6%以上，公共财政预算支出增长15%；非税收入占比下降5个百分点以上；2000万元以上工业增加值增长13%以上；50万元以上固定资产投资增长25%以上；招商引资实际到位资金增长21%以上；社会消费品零售总额增长15%以上；金融机构存贷款余额分别增长11%、17%以上；城镇居民人均可支配收入增长12%；农民人均可支配收入增长14%；人口出生率控制在12.41‰以内，人口自然增长率控制在5.61‰以内；城镇登记失业率控制在4.2%以内；完成省下达的节能减排指标任务。

　　各位代表！做好今年的工作，确保完成上述目标任务，对于“十二五”圆满收官、“十三五”良好开局至关重要。我们将围绕适应新常态、抢抓新机遇、实现新作为，精心编制好“十三五”规划，为同步小康谋好篇、布好局。我们将依靠全面深化改革和全面推进依法行政，解决新常态下面临的各种困难和问题，用法治引领改革发展破障闯关，用改革推动法治建设破浪前行，共同推动全面建成小康社会事业滚滚向前。

　　全面深化改革方面，更加突出问题导向和需求导向，以切实可行的改革方案、落地见效的改革措施，推进重点领域改革。深化投融资体制改革。制定落实国家和省创新重点领域投融资机制鼓励社会投资的实施意见。全面统筹项目融资需求，规范过渡期融资工作。大力推行PPP等模式，积极引入社会资本，完善借、管、用、还一体化财政金融联动机制。深化实体经济领域改革。完善先照后证制度，积极争取“电子执照”试点。出台加快国有企业发展混合所有制经济政策措施。建立“政、银、企”三方联席会议制度，引导金融机构加大对实体经济支持力度。做好公共资源交易互联互通试点工作，规范完善公共资源交易目录范围。深化财税体制改革。深入推进“营改增”，认真组织实施煤炭资源税从价计征改革。深化预算管理制度改革，建立跨年度预算平衡机制。制定完善市县两级财政管理综合绩效评价体系。清理和规范税收优惠政策。深化统筹城乡发展体制改革。深入推进城乡规划“多规融合”。开展土地征收、集体经营性建设用地入市、宅基地制度改革试点。加快推进土地承包经营权确权登记颁证。全面完成乡镇行政区划调整。完善扶贫项目资金使用管理机制和贫困乡镇考核办法。深化生态文明建设体制改革。启动北盘江流域生态文明改革试点。制定生态建设环境保护指标体系和考核办法。出台环境污染第三方治理实施细则。制定环境重点案件督导督办制度和最严格的环境影响评价制度。深化社会领域综合改革。推进教育综合改革。全面实施县级公立医院综合改革。制定实施机关事业单位养老保险改革方案。加快推进公交体制改革。建立和完善社区网格化管理工作机制。

　　全面推进依法行政方面，深入贯彻依法治国基本方略，大力推行依法行政、从严执政，加快建设法治政府。依法履行政府职能。坚持法定职责必须为、法无授权不可为。对法定职责范围内的行政事务，勇于负责、敢于担当，坚决纠正不作为、乱作为，坚决惩处失职、渎职。推行政府权力清单、责任清单和负面清单制度。大力推进“减政、减负、减支”，扎实抓好政务服务、公共资源交易、电子政务平台建设。深化简政放权，全力做好行政审批“接、放、管”各项工作。健全依法决策机制。决策必须符合民意民心。把公众参与、专家论证、风险评估、合法性审查、集体讨论决定作为重大行政决策法定程序，确保政府决策合法合规。全面推行政府法律顾问制度。坚持政府重要文件合法性审查。建立重大决策终身责任追究制度及责任倒查机制。完善决策咨询机制，注重听取各民主党派、工商联、无党派人士和各人民团体的意见建议。严格公正文明执法。坚持猛药治疴、重拳治乱，让法治成为政府全体公职人员的信仰。大力推进行政处罚、行政强制、行政征收、行政检查等规范化执法，积极推进综合执法。严格实行行政执法人员持证上岗、资格管理和行政执法全过程记录和公示制度。加强执法人员教育和培训，提升执法能力和水平。强化权力监督制约。自觉接受人大法律监督、政协民主监督，主动接受司法监督、新闻媒体监督和社会公众监督。强化政府内部层级监督和专门监督，完善行政权力运行机制，真正把权力关进制度的笼子。依法推进政务公开，让权力在阳光下运行。严肃问责，严厉惩处权钱交易等腐败行为。加大廉政建设力度。巩固党的群众路线教育实践活动成果，坚决落实中央八项规定、国务院“约法三章”和省委、市委十项规定，持续深入反“四风”改作风。自觉践行“三严三实”，全面落实党风廉政建设主体责任，严明党的各项纪律，建设廉洁政府。

　　三、2015年重点工作

　　围绕实现全年目标，重点抓好以下工作：

　　（一）围绕扩投资促消费，全力保持经济稳定增长。充分发挥投资的支撑作用和消费的拉动作用，确保50万元以上固定资产投资完成1670亿元、社会消费品零售总额完成272亿元。

　　着力扩大有效投资。抢抓国家实施“七大投资工程包”机遇，努力把政策机遇变成项目和资金。调整优化投资结构。注重调结构、补短板、惠民生，多管齐下扩大有效投资，财政建设性投资重点用于支持培育新的经济增长点。完成工业投资750亿元、农业投资147亿元、城镇建设投资261亿元、房地产开发投资200亿元、交通投资90亿元、水利投资62亿元、社会事业投资160亿元。注重扩大产业投资，产业投资比重保持在45%以上。强化项目建设抓手。做深做实项目前期工作，继续实行项目集中开工机制，全年集中新开工项目1200个以上，完成投资800亿元以上。推行项目观摩机制。加强项目调度督查，推进签约项目抓落地、落地项目抓建设、建设项目抓投产。加强投资要素保障。大力争取上级项目资金支持，积极争取金融机构信贷资源倾斜，着力解决项目资金难题。转变土地利用方式，提高土地投入产出水平，满足项目建设需求。加强用水、用电、用工、拆迁安置等各项保障，全力做好协调服务工作。有效防范经济风险。全面落实新预算法，加强地方政府性债务清理和化解。在基础设施及公共服务等领域推广PPP、BOT等模式，提高资产经营水平。密切关注煤矿、房地产、金融等行业的潜在风险，有效防控经济问题引发的社会风险。严打非法集资，净化金融市场环境。

　　着力促进消费增长。大力培育消费热点。积极争取国家“六大领域消费工程”项目支持，利用独特的气候、资源、区位等优势，大力培育健康、养老、旅游、信息、住房、绿色、教育文化体育消费，满足群众个性化、多样化消费需求。加快完善市场体系。积极培育壮大流通主体，大力引进国际国内知名零售商。加强农村商贸流通设施建设，积极发展农村电子商务，新建和改造一批配送中心和“万村千乡市场工程”农家店。有效促进大众消费。鼓励和引导商贸流通企业依法开展形式多样的促销活动。大力发展会展经济。加强市场秩序整顿和规范，营造良好的消费环境。

　　（二）围绕优结构增后劲，全力推进产业转型升级。按照市场要活、创新要实、政策要宽的要求，坚定不移地推进一产转型、二产升级、三产优化。

　　围绕“百姓富、生态美”推动一产转型。立足资源禀赋和市场需求，打好“生态牌”、念好“山字经”、种好“摇钱树”，加快转变农业发展方式，着力打造喀斯特山区特色农业示范区，把山地特色农业培育成为新的经济增长点。大力发展农业特色产业。按照产业生态化、生态产业化的要求，大力推进农业结构调整，完成农业特色产业“3155工程”种植85.5万亩。扎实推进苗木基地建设，完成苗木培育基地1万亩。坚持种管并重，建立后续管护机制。推进品种更新、品质提升、品牌创建，大力打造凉都圣果、凉都红豆杉、凉都春芽、凉都核桃、凉都刺梨、凉都红樱桃等特色农产品品牌。加强农业科技队伍能力建设，积极与省内外农科院（所）和农业院校合作，建立特色产业研发中心和研发基地。加快推进现代高效农业示范园区建设。提升现代高效农业示范园区规模和质量，新增1个以上发展型园区为引领型园区，新增1个以上市级园区为发展型园区，高标准建成六枝郎岱、盘县滑石、水城米箩、钟山大河等8个示范园区。大力发展特色有机农业、生态农业、旅游农业，走产出高效、产品安全、资源节约、环境友好的现代农业发展之路。创新农业经营体系。积极推动农业适度规模经营，重点培育农民合作社、专业大户、家庭农场等各类新型农业经营主体；引导本地企业转型发展特色农业，引进、培育一批农产品精深加工龙头企业；鼓励和支持返乡大学生、乡土人才、返乡创业人员创办示范园、示范场和农副产品加工企业；大力培育农村经纪人队伍。分别新增省级、市级重点龙头企业10家、25家以上。加快供销合作社改革发展。加大农业发展支持力度。以县为单位加大涉农项目资金整合力度，健全金融支农制度，发展农业融资担保和再担保机构，扩大农业政策性保险覆盖面。按照“产权变股权、资金变股金、农民变股民”的要求，加快发展壮大村级集体经济，盘活村级资金、资产和资源，丰富村级集体经济业态。

　　围绕“结构好、效益高”推动二产升级。大力实施工业“百千万”工程。坚持盘活存量“过日子”，以实施工业“百千万”工程为抓手，政企同力、抱团取暖，分类指导、一企一策，推动煤炭、电力、钢铁、铝业、建材等传统工业企业开足马力。支持鼓励传统工业企业瞄准市场，优化产品结构，推动技术创新，提升产品市场竞争力。加快推进煤矿企业兼并重组，推动煤炭行业转型升级，煤炭资源就地转化率提高10个百分点以上。科学统筹煤电化、煤电材一体化产业园区建设，支持黔桂天能大力发展精深煤焦化产业。推进上海华谊集团与水城矿业集团深度合作，创造条件帮助路喜园区焕发活力，大力支持六枝工矿、盘江控股、水城矿业集团加快改革改制工作。坚持提升增量“强底子”，大力推进产业园区建设，完善水、电、路、气和绿化等基础设施。加快一批园区项目建成投产，尽快释放产能，提供增量支撑。重视产业园区要素资源聚集和自我发展能力建设。大力发展循环经济。高举循环经济旗帜，把循环经济培育成为新的经济增长点。力争国家发展改革委年内批复《六盘水市建设国家循环经济示范城市发展规划》。大力实施循环经济园区、产业、企业、项目，全力推动六枝工矿、盘江控股、水城矿业集团加快传统煤业和现代煤化工一体化发展，加快水钢循环经济轻型墙体材料和钢材深加工项目建设，确保黔桂天能煤焦化下游产品开发实现25个以上。加快推进煤层气、页岩气和再生能源的开发利用，加大粉煤灰、煤矸石等工业固体废弃物的综合利用，推进紫森源综合利用项目完成二期中试。大力发展新兴产业。创造条件发展以大数据为核心的电子信息产业，支持钟山区呼叫中心产业园、盘县LED产业园、红桥梅安森等企业发展，加强与富士康深度合作。充分发挥中草药资源和凉都气候资源优势，加快发展大健康医药养身产业，支持红豆药业、汉方集团、三特药业、宏奇药业、圣济堂药业等做强做大；规划建成一批万亩中药材基地，推进道地中药材原产地认证；积极引进知名药业集团到我市投资兴业。加快发展新型建筑建材产业，加快建设一批新型建材产业基地，支持安凯达等企业研发新型建筑材料。整合各类建筑公司资源，集中力量培育壮大一批建筑业龙头企业，支持乡村建筑队伍做强做大，提升本土建筑行业的资质和实力。

　　围绕“市场活、可持续”推动三产优化。立足融合发展抓好生产性服务业。加强物流通道、物流网络、物流园区和物流企业培育，重点推进红桥物流园区、月照机场临空产业园、六盘水南方煤炭储备交易中心等建设。大力发展现代商务、研发设计、检验检测、节能环保等生产性服务业。加快发展电子商务；推进六盘水电子商务产业园和西南盘兴电子商务公司建设，争取列入“国家级电子商务示范企业”。加大“引金入市”力度，支持各类金融机构在六盘水设立分支机构和后台服务机构，力争引进2家以上银行在我市挂牌运营。立足提升水平抓好生活性服务业。围绕建设大健康旅游目的地城市，加大“贵州屋脊·中国凉都”品牌宣传推介力度，加快构建“快旅慢游”服务体系，打造六盘水文化旅游发展升级版。全年旅游收入增长30%以上。完成旅游业发展总体规划编制，加快编制各景区景点专项规划；加快完善旅游基础设施和配套设施，建成野玉海1至6号路等一批景区公路，完成四格至毛口旅游精品线立项并争取年内开工建设，每个县区打造1个以上“四在农家·美丽乡村”升级版示范点。大力发展以健康服务和养老服务为重点的休闲地产、旅游地产；着力打造旅游精品，加快牂牁江、廻龙溪、妥乐古银杏、乌蒙大草原、野玉海、玉舍滑雪场、韭菜坪、水城古镇等重点旅游景区深度开发，开工建设梅花山国际生态休闲度假区，建成海坪等一批旅游小镇，大力发展特色工艺品、特色美食等旅游商品；提升旅游管理服务能力，提供人性化、个性化、精细化服务。加快发展家政、社区服务等服务业。

　　（三）围绕城乡统筹发展，全力推进山地特色新型城镇化。坚持以人为本、道法自然，完善思路、创新举措，走山地特色新型城镇化路子。

　　加快推进“三地”同城。坚持规划引领。按照产城景互动和“多规合一”的要求，做好“三地”各类规划的衔接和融合；编制完成《市中心城区控制性详细规划》和生态保护、水系、景观、道路、供气等专项规划。加快建设数字规划信息管理平台，实现规划同城统一。严格规划执法，保持对“两违”建筑的高压态势。坚持基础先行。开工建设一批“三地”同城路网，确保机场高速上半年建成；红桥东路、康乐南路、麒麟路、德宏路年内建成。争取以PPP模式推进城市轨道交通立项和开工。加快推进棚户区改造，全面实施今年68101户改造任务。统筹推进供水、供电、供气等设施建设，提升城市承载能力。鼓励社会资本参与投资、建设和运营城市基础设施项目。坚持管理提升。深入开展“四创”活动，按计划推进各项创建活动，推动活动向县乡村延伸覆盖。进一步完善城市管理机制，提升城市管理精细化、信息化、规范化水平。

　　加快特色示范小城镇建设。着力推动产城景互动。坚持把小城镇建设与产业园区、旅游景区景点等有机结合起来，打通城区、园区和景区的通道，实现以城聚产、以产兴城、产城景互动融合。着力完善功能配套。按照“政府主导、企业参与、市场运作”的原则，加大财政专项资金投入，大力开展招商引资，推动特色城镇建设。深入实施“8+X”项目，加强供水、供电、排污、环卫等基础设施建设，完善教育、卫生、文化、体育等公共服务配套，提升城镇承载能力。着力打造特色亮点。统筹山水、田园、乡村、城镇等要素，彰显自然景观、建筑风格、民族风情和文化品位，启动10个以上特色小城镇建设，新增省级、市级示范小城镇各1个以上。把柏果镇、岩脚镇、郎岱镇、玉舍镇建设成为省级“样板镇”。

　　加快美丽乡村建设。坚持规划统筹、把握标准、整合资源、产业跟进的原则，深入实施“四在农家·美丽乡村”六项行动计划，以县乡为主体，整乡整村推进，集中连片打造，扩大覆盖面和受益面，让美丽乡村处处可见。突出抓好资金筹措。统筹整合好各类资源要素，大力争取上级资金，积极引导社会资金投入，拓宽融资渠道，扩大融资规模。突出抓好建设管理。建立“建、营、管”一体化机制，确保各项设施正常运转、永续使用。坚持政策、项目、对象、投入、补贴“五个公开”，强化资金监管，及时足额兑现。严格审计和监督，严肃查处违纪违法行为。突出抓好产业培育。大力发展特色种养业和乡村旅游业，让更多的特色农产品走出乡村、走向市场，让“美丽乡村”生态美、产业美、环境美。

　　（四）围绕改善发展条件，全力加快基础设施建设。加快现代综合交通体系建设。深入实施交通建设“三年会战”，加快推进沪昆客运专线境内段和安六城际铁路建设，加快水城至盘县城际铁路、盘县松河至响水运煤专线前期工作。杭瑞高速境内段建成通车，加快盘兴高速建设，开工建设都香高速六威段，加快纳晴高速境内段前期工作，争取六盘水至宜宾高速纳入国家和省发展规划。完成国省道改造97公里；开工建设一批重点区域干线公路、旅游公路、运煤公路。加快农村公路管护体制改革。加强月照机场运营管理，开通更多航班航线；推动盘县支线机场纳入国家民航发展空间布局规划。积极配合省实施好光照库区航运建设工程，力争北盘江航运建设工程纳入《西江航运干线航道建设规划》。加快现代水利工程网络建设。深入实施水利建设“三大会战”，力争纳入“三位一体”规划的水源点年内全部开工建设，加快推进朱昌河、懒龙河等中型水库建设，建成卡河水库、红岩水库等骨干水源工程；大力推进六枝、钟山小型农田水利重点县及盘南中型灌区建设；加强地下水开发利用，切实解决好工程性缺水问题。加快能源信息基础设施建设。完成电网投运35千伏及以上变电站5座，线路431公里；改造数字化变电站3座、投运2座。开工建设中缅天然气管道六盘水支线工程和六枝天然气液化项目，完成红桥LNG输气站建设。加快实施信息基础设施建设“三年会战”，全力推进“宽带六盘水”等重点工程建设。加快智慧城市创建。

　　（五）围绕建设美丽凉都，全力加快生态文明建设。以“绿色贵州”三年行动计划为契机，坚持造林绿化与石漠化治理、产业发展、改善环境、景观打造相结合，把生态做成产业、把产业做成生态，努力实现生态价值、经济价值、社会价值、旅游价值最大化，把生态产业培育成为新的经济增长点。着力在“建”上求突破。以“3155”特色产业为载体，以退耕还林还草和石漠化治理项目为支撑，用三年时间集中完成荒山绿化、退耕还林、石漠化治理150万亩。森林覆盖率每年增加3个百分点以上。着力在“治”上下狠劲。下大力治理水环境，深入实施重点流域河长制，建成市污水处理厂工程，按标准改造提升8个、新建6个城镇污水处理厂，城市污水处理率达到87%；下大力防治大气污染，扎实开展大气污染防治行动计划，确保首钢水钢4#、5#烧结机及六枝工矿、盘江控股、水城矿业集团矸石发电厂主要污染物超标排放通过整改验收，提高城市空气质量优良率；下大力解决废渣问题，加快推进水城县海螺水泥和盘县三合水泥窑协同处理垃圾、钟山区垃圾焚烧发电等项目，城市生活垃圾无害化处理率达到75%以上。扎实做好节能减排工作，全面完成“十二五”节能减排指标任务。着力在“管”上出实招。深入贯彻新环境保护法，用好森林保护“六个严禁”、环境监管“六个一律”两把“利剑”，强化林业生态红线保护和环境污染“两个问责”，严肃查处生态环保不作为、乱作为行为，让“美丽凉都拒绝污染”深入人心。

　　（六）围绕激发动力活力，全力实施开放创新。抓住国家推进新一轮高水平对外开放带来的新机遇，全面提高开放带动和创新驱动能力。搭建开放带动平台。用好生态文明贵阳国际论坛六盘水分论坛、凉都消夏文化节两个平台。加快推进临空经济区、“无水港”等载体建设，力争国家尽快批复成立海关和检验检疫机构。瞄准黔中、滇中经济区，深化与泛珠三角区域合作，更好更快融入“一带一路”、长江经济带和珠江—西江经济带，全力打造“双向桥头堡”。加强与瑞士、台湾、大连等区域交流合作，推进毕水兴和周边区域经济合作。打造创新驱动引擎。大力实施“六大科技行动”，启动红桥、六枝、红果、水城经济开发区科技企业孵化器和6个市级以上重点实验室、工程技术研究中心项目建设。加强知识产权优势企业培育，形成多项关键技术专利或专利群。深化与省科技厅的科技合作，争取紫森源、安凯达等企业重大专项项目申报工作取得突破。制定切实可行的企业、项目科技创新目标体系，力争更多科技创新项目进入省级以上规划和实施计划。大力发展民营经济。坚持企业为基，做多、做强、做精市场主体，全年新增市场主体1.8万户，新增注册资金200亿元。深入推进民营经济五年行动计划，完成民营经济增加值660亿元、增长22.5%，完成民间投资780亿元、增长25.8%。着力破解民营企业准入难、融资难、政策落地难等问题，鼓励民营企业进入交通运输、信息产业、能源、水利、教育等领域，支持社会资本参与国有企业改革；搭建民营企业金融服务平台，创造条件提供更多信贷支持；坚决落实国家、省和市政策措施，让民营企业在政策阳光的普照下茁壮成长。

　　（七）围绕提升公共服务，全力发展各项社会事业。把繁荣发展社会事业作为促进社会公正、增进人民福祉的重要途径。优先发展教育事业。深入实施教育“9+3”计划，继续压缩行政经费5%用于教育“9+3”计划。大力实施基本普及15年教育计划。加强农村寄宿制学校标准化建设和管理。加快高中教育城、以朵职教城建设。推进六盘水师院二期、六盘水职院二期改扩建工程建设，确保六盘水师院通过教育部本科教学合格评估，六盘水职院顺利通过省级示范评估验收。加快发展各级各类教育。大力发展卫生计生事业。加快凉都人民医院建设。加强基层卫生计生机构标准化和全科医生队伍建设。加强与国内外知名医疗院所合作，大力发展优质医疗。突出抓好人口计生服务管理创新，扎实开展严打“两非”专项治理行动，有序推进“单独二孩”生育政策和计生利益导向“四项制度”落实，确保实现人口计生“三降一升”目标。加快发展文化体育事业。抓住省实施“八大文化惠民工程”机遇，推进现代公共文化服务体系建设。高标准、高质量办好凉都消夏文化节系列活动。加强文化遗产保护。加快老王山生态型多梯度高原运动训练示范基地大本营一期、野玉海耐力性高原训练基地建设。统筹发展其他社会事业。扎实推进殡葬制度改革，加快推进西福园公墓和市殡仪馆迁建项目建设。深入实施《全民科学素质行动计划纲要》，提高市民科学素质。重视国防建设，支持军队、武警部队建设和法院、检察院工作。加强国家安全、司法行政、双拥优抚、民族、宗教、人防、档案、外事、侨务、老龄、对台、气象、工青妇、残疾人、地方志、红十字等工作。

　　（八）围绕增加民生福祉，全力保障和改善民生。按照守住底线、突出重点、完善制度、引导舆论的思路，切实做好改善民生各项工作。实施扶贫攻坚。深入推进片区扶贫、整体扶贫、党建扶贫、社会扶贫、对口扶贫，做到精准扶贫“六个到村到户”，贫困人口发生率降低到17%以下。实行目标、任务、资金和权责“四到县”制度，整合用好扶贫资金，加强扶贫资金审计。努力扩大就业。加快推进国家级创业型城市创建工作，实施全民创业行动计划和“双百工程”，推动大众创业。积极帮助困难群众和特殊人群就业。健全社会保障。进一步扩大基本养老、基本医疗、失业、工伤、生育保险覆盖面，实施全民参保登记计划，全市基本社会保险参保覆盖率达到90%以上；城乡低保标准提高10%以上；推进市社会福利中心迁建工程。认真落实临时救助制度。加强住房保障。推进城镇保障性住房、农村危房改造和扶贫生态移民工程建设，规范廉租房、公租房、棚改房分配，强化资金管理和使用。开工建设保障性住房4378套，完成农村危房改造1.5万户。加快推进政策性农房灾害保险工作。狠抓安全生产。认真落实新安全生产法，深入开展煤矿安全教育实践活动，进一步强化红线意识、底线意识，全面落实“8个100%”措施，确保煤矿安全生产形势持续稳定好转。深入开展“六打六治”和隐患排查治理等专项行动，统筹抓好道路交通、非煤矿山、建筑施工、危化物品、地质灾害、消防等领域安全生产工作。加强食品药品安全监管。创新社会治理。深入推进“平安凉都”建设，坚决打好命案攻坚、禁毒严打整治、打击涉黄涉赌违法犯罪“三大战役”。强化公共安全监管和应急处置体系建设，排查化解矛盾纠纷和信访问题，营造平安和谐稳定环境。

　　扎实办好十件民生实事。（1）创建市级示范中小学校（幼儿园）15所；新建、改扩建13所公办幼儿园；推进农村义务教育25万学生营养改善计划；治理受地质灾害危害学校27所。（2）改扩建乡镇卫生院13个，其中，重点乡镇卫生院12个，一般乡镇卫生院1个；开工建设六盘水市人民医院全科医生规范化培训培养基地；改建村卫生室100个。（3）新增新能源公交车20辆、出租车350辆。（4）实施扶贫生态移民工程搬迁1800户7100人；完成9个贫困乡镇“减贫摘帽”，实施50个贫困村“整村推进”。（5）继续实施“3个15万元”扶持政策，新增微型企业1300户，带动就业6500人以上；城镇新增就业9.24万人。（6）解决全市23.53万农村人口饮水安全问题；确保县城及乡镇千人以上集中式饮用水源地水质达标率100%。（7）完成10所农村敬老院改造提升。（8）加强城市配套设施建设，建成人民广场及钟山大道部分地下人防工程；建成10个地下人行通道；新建城市停车场1个（495个停车位）；实施市中心城区集中供热100万平方米；建成六盘水污水处理厂。（9）基本完成水城河综合治理工程。（10）大力推进绿色贵州六盘水造林绿化工程，完成荒山绿化、石漠化治理、退耕还林50万亩。

　　各位代表！凉都大地春潮涌，扬帆破浪正当时。让我们紧密团结在以习近平同志为总书记的党中央周围，在省委、省政府和市委的坚强领导下，借势扬势，担当作为，让凉都经济热起来，让凉都环境美起来，让凉都人民富起来，为全面完成“十二五”规划目标任务、创造六盘水更加幸福美好未来而努力奋斗！
